# Supplementary material for: Lack of tetrodotoxin analogues and individual metabolomic profiling of the cryptic frog Colostethus imbricolus
Source: PLoS One. 2026 Apr 16;21(4):e0325877. doi: 10.1371/journal.pone.0325877 (PMC13086437; doi:10.1371/journal.pone.0325877)

**Lack of tetrodotoxin analogues and individual metabolomic profiling of the cryptic frog *Colostethus imbricolus***

Mabel Gonzalez^12^, Pablo Palacios-Rodriguez^3^, Chiara Carazzone^1^*

^1^ Department of Chemistry, Universidad de los Andes, Bogotá, Colombia

^2^ Department of Biology, Stanford University, Palo Alto, CA, EEUU

^3^ Facultad de Estudios Ambientales y Rurales. Pontificia Universidad Javeriana, Bogota, Colombia

*Corresponding author

E-mail: [c.carazzone@uniandes.edu.co](mailto:c.carazzone@uniandes.edu.co)

**Resumen:** Las ranas venenosas (Dendrobatoidea) se caracterizan por la gran diversidad de alcaloides descubiertos en su piel. Sin embargo, la mayoría de estos alcaloides se han encontrado en especies de coloración conspicua y existe un gran desconocimiento de los perfiles de alcaloides en las especies menos coloridas. Tetrodotoxinas paralizantes (TTXs) se han reportado previamente en dos especies de coloración críptica del género *Colostethus*, y estas son las dos únicas ocurrencias de alcaloides hidrofílicos en la superfamilia Dendrobatoidea. Resultados no publicados utilizando extractos de *Colostethus imbricolus* inyectados intraperitonealmente en ratones, demostraron que esta especie contiene sustancias paralizantes. Para analizar los metabolitos de la piel y determinar si corresponden a TTX, o a análogos de TTX, hemos empleado una separación dirigida a TTX en gradiente de fase normal, y un perfilamiento no dirigido en gradiente de fase inversa. Tras realizar ambos análisis, no se detectaron ni TTX ni análogos de TTX en *C. imbricolus*. Por el contrario, se separaron otros metabolitos, permitiendo la extracción de 76 aductos comunes a ambos análisis, siendo 33 de ellos anotados tentativamente como alcaloides de anfibios, ocho como metabolitos de anfibios diferentes de alcaloides y 25 que coinciden con productos naturales del DNP. Un total de 10 fórmulas moleculares comunes no pudieron ser anotadas. La ausencia de espectros MS/MS para estos aductos requiere una futura confirmación de sus estructuras, una vez se complete la adquisición dirigida por MS/MS. Al analizar la variación interindividual de seis especímenes, se demostró que el metaboloma de la piel difiere entre machos y hembras de *C. imbricolus*. De esta forma, nuestros resultados permiten concluir que la TTX no es el único compuesto paralizante en dendrobatidos y que deben realizarse más estudios enfocados a entender este fenómeno. Un resultado adicional de este estudio es la primera separación exitosa de TTX en una columna SB-CN utilizando un gradiente de fase normal, lo que permite un método novedoso para la separación dirigida de TTX.

Figure S1. Graphical abstract from this study. Created in [https://BioRender.com](https://biorender.com)

Figure S2. Base peak chromatograms (BPC) (to the left) and the corresponding MS/MS fragmentation patterns (to the right) from target masses of interest in the preliminary analysis of *C. imbricolus*. Two instrumental replicates of the same sample are presented for each ion (r001 and r002). The most intense molecular feature (178.1342) and eight target masses of TTX analogues (3 pages). *The 20-minute gradient on the SB-CN column was employed in these analyses.


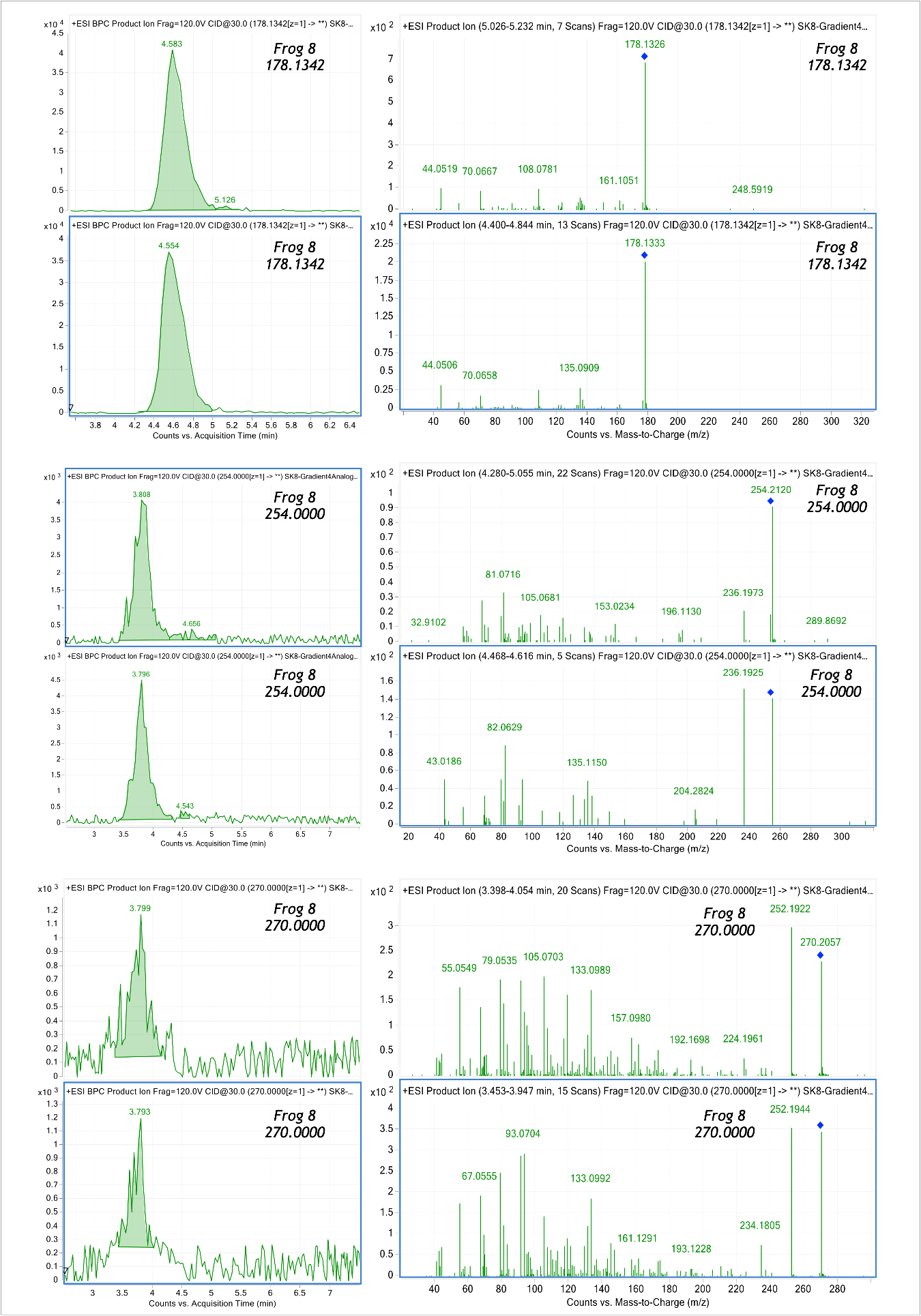


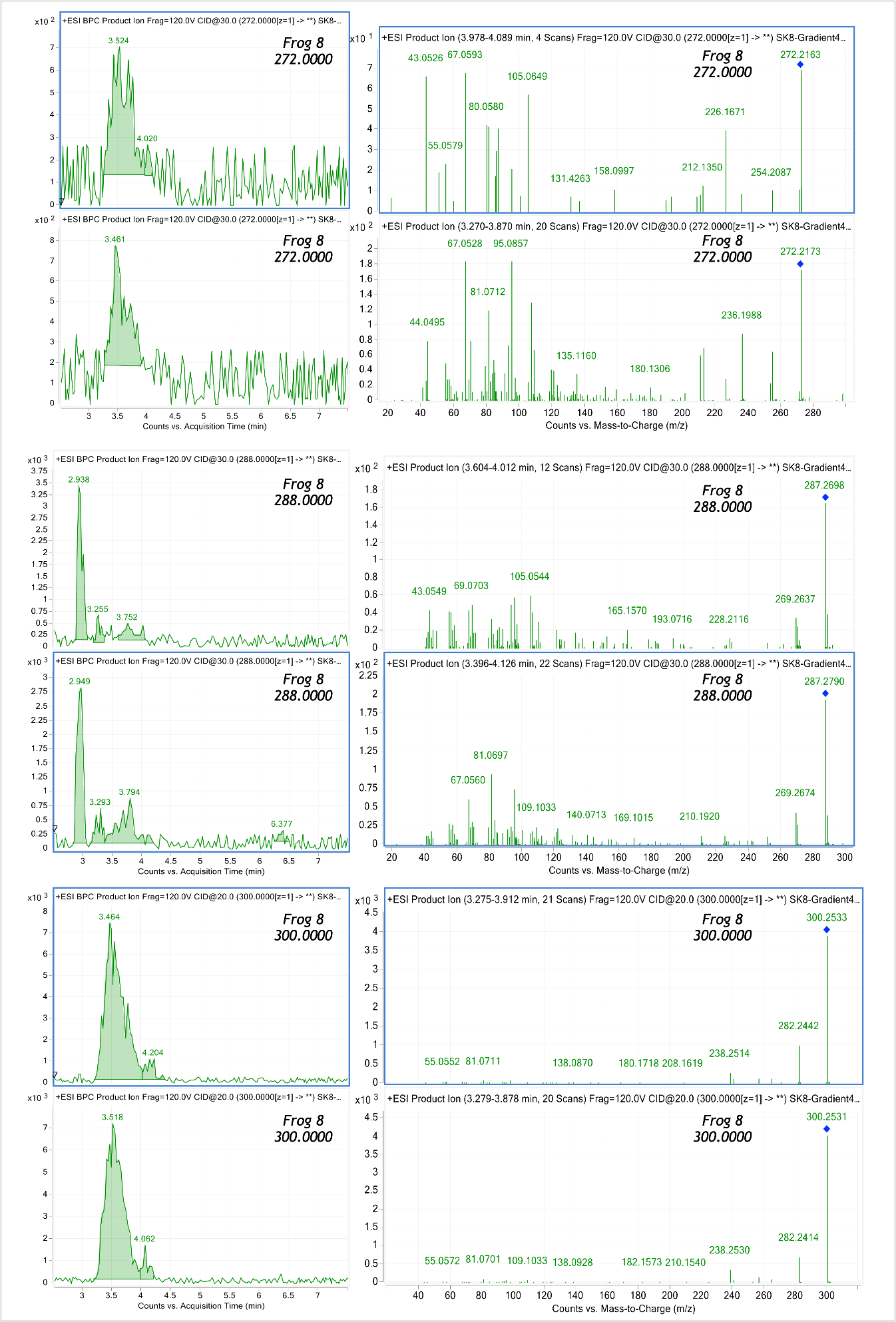


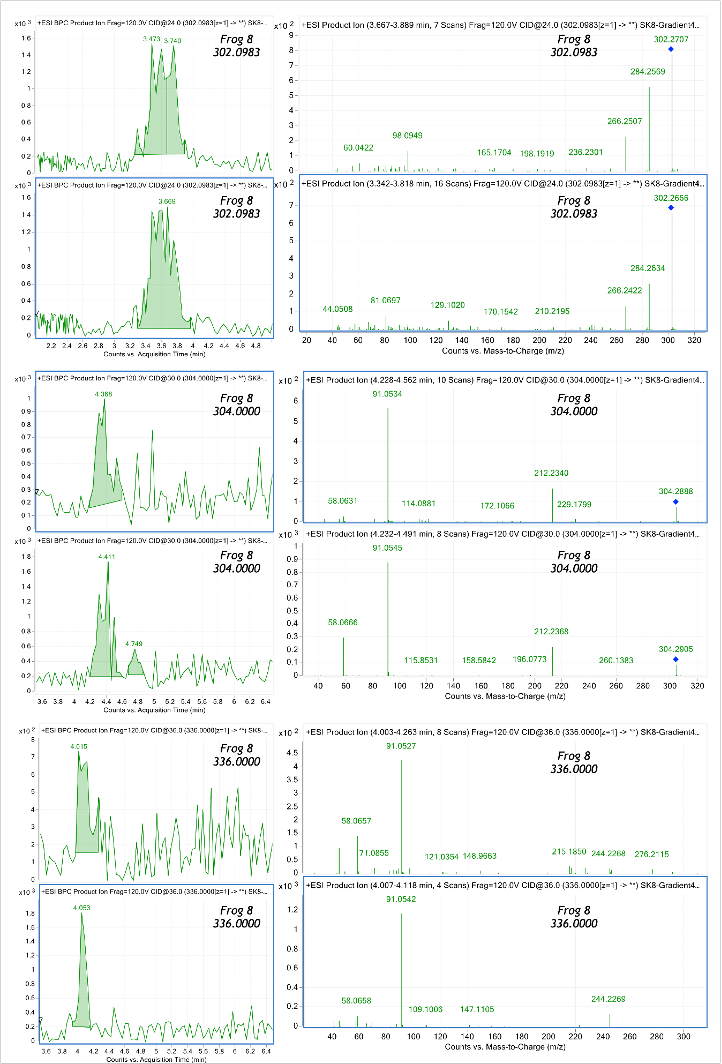


Figure S3. Chromatographic separation of TTX employing a chromatographic method of 20 minutes in SB-CN column. A. Extracted ion chromatogram (EIC) from accurate mass of TTX (320.1088) in a blank of MeOH and three instrumental replicates of 10 ppm TTX solutions. B. Verification of deconvolution process of TTX molecular formula prediction. C. MS/MS fragmentation pattern of TTX. r001, r002 and r003 correspond to instrumental replicates of the same sample.


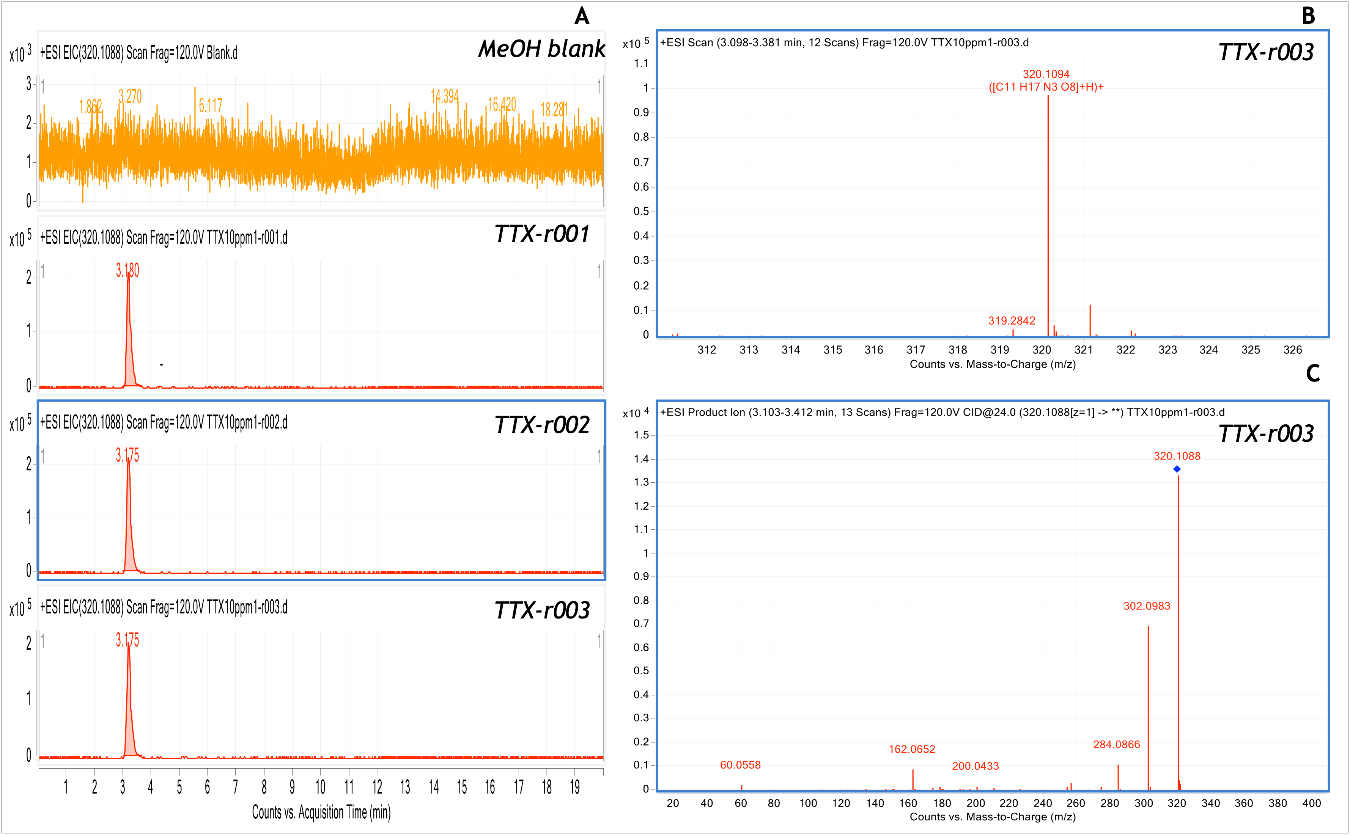

Supplement: S1 File — (DOCX) [file pone.0325877.s002.docx]
