# Supplementary figures and images for: Lack of tetrodotoxin analogues and individual metabolomic profiling of the cryptic frog Colostethus imbricolus
Source: PLoS One. 2026 Apr 16;21(4):e0325877. doi: 10.1371/journal.pone.0325877 (PMC13086437; doi:10.1371/journal.pone.0325877)

**Graphical abstract**

**
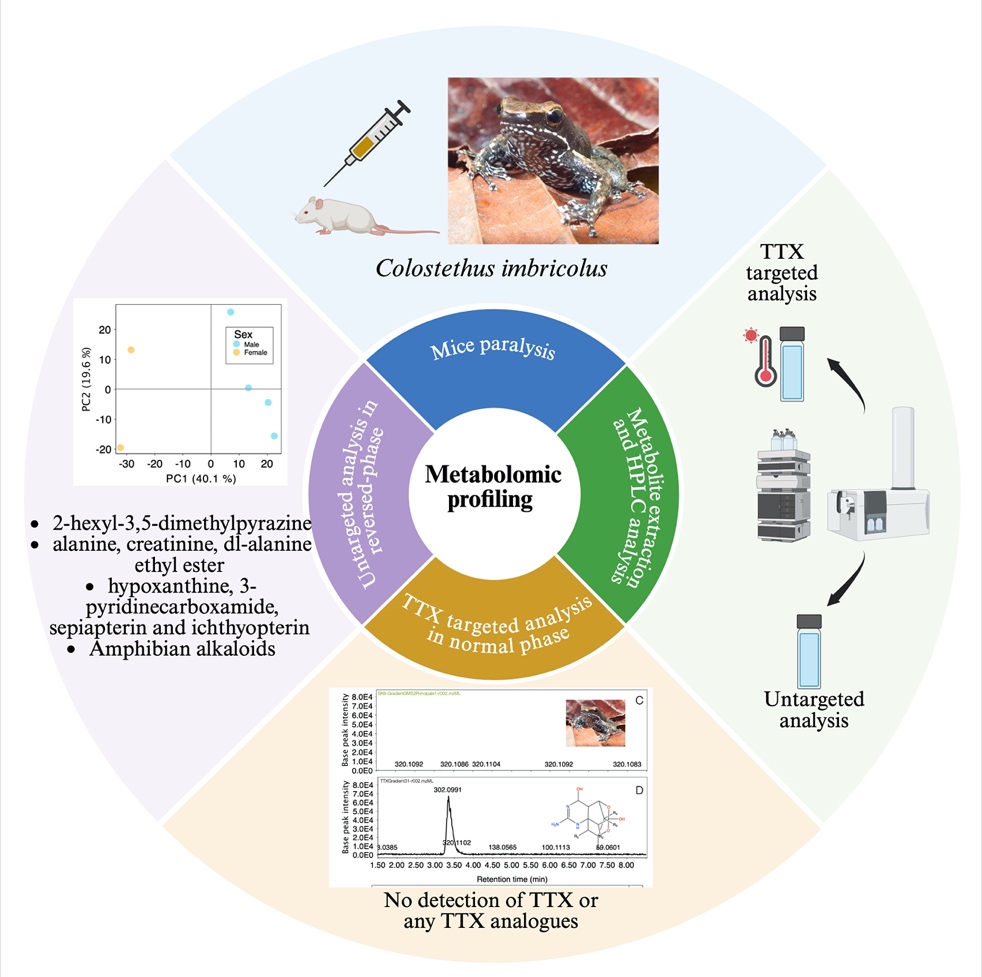
**

Created in BioRender. Su, L. (2025) <https://BioRender.com/zyeybl6>

Supplement: S1 Fig — (DOCX) [file pone.0325877.s007.docx]
