# Supplementary material for: Lack of tetrodotoxin analogues and individual metabolomic profiling of the cryptic frog Colostethus imbricolus
Source: PLoS One. 2026 Apr 16;21(4):e0325877. doi: 10.1371/journal.pone.0325877 (PMC13086437; doi:10.1371/journal.pone.0325877)
Supplement: Attachment — Submitted filename: Response reviewers- PLOSONE-Colostethus.docx [file pone.0325877.s009.docx]

**Journal Requirements:**

When submitting your revision, we need you to address these additional requirements.

1. Please ensure that your manuscript meets PLOS ONE's style requirements, including those for file naming. The PLOS ONE style templates can be found at

https://journals.plos.org/plosone/s/file?id=wjVg/PLOSOne_formatting_sample_main_body.pdf and <https://journals.plos.org/plosone/s/file?id=ba62/PLOSOne_formatting_sample_title_authors_affiliations.pdf>

***Response.*** We have corrected the format for author affiliations.

2. Thank you for stating in your Funding Statement:

[The research was supported by the announcement No. 757–2016 Doctorados Nacionales and project contract No. 44842–058-2018 from Ministerio Administrativo de Ciencia, Tecnología e Innovación (MINCIENCIAS). The financial support from the Faculty of Science at Universidad de los Andes partitioned in a forgivable loan assigned to one doctoral student (M.G.), the seed projects INV-2018-33-1259, INV-2019-67-1747 and FAPA project of C.C. The authors would like to thank the Vice Presidency of Research & Creation’s Publication Fund and the Science Faculty at Universidad de los Andes for its financial support too.].

Please provide an amended statement that declares *all* the funding or sources of support (whether external or internal to your organization) received during this study, as detailed online in our guide for authors at http://journals.plos.org/plosone/s/submit-now. Please also include the statement “There was no additional external funding received for this study.” in your updated Funding Statement.

Please include your amended Funding Statement within your cover letter. We will change the online submission form on your behalf.

***Response.*** We have edited the Funding Statement following your instructions and included this text within the cover letter.

3. Thank you for stating the following in your manuscript:

[The research was supported by the announcement No. 757–2016 Doctorados Nacionales and project contract No. 44842–058-2018 from Ministerio Administrativo de Ciencia, Tecnología e Innovación (MINCIENCIAS). The financial support from the Faculty of Science at Universidad de los Andes partitioned in a forgivable loan assigned to one doctoral student (M.G.), the seed projects INV-2018-33-1259, INV-2019-67-1747 and FAPA project of C.C. The authors would like to thank the Vice Presidency of Research & Creation’s Publication Fund and the Science Faculty at Universidad de los Andes for its financial support for the article processing charge.]

We note that you have provided funding information that is not currently declared in your Funding Statement. However, funding information should not appear in the Acknowledgments section or other areas of your manuscript. We will only publish funding information present in the Funding Statement section of the online submission form.

Please remove any funding-related text from the manuscript and let us know how you would like to update your Funding Statement. Currently, your Funding Statement reads as follows:

[The research was supported by the announcement No. 757–2016 Doctorados Nacionales and project contract No. 44842–058-2018 from Ministerio Administrativo de Ciencia, Tecnología e Innovación (MINCIENCIAS). The financial support from the Faculty of Science at Universidad de los Andes partitioned in a forgivable loan assigned to one doctoral student (M.G.), the seed projects INV-2018-33-1259, INV-2019-67-1747 and FAPA project of C.C. The authors would like to thank the Vice Presidency of Research & Creation’s Publication Fund and the Science Faculty at Universidad de los Andes for its financial support too.]

Please include your amended statements within your cover letter; we will change the online submission form on your behalf.

***Response.*** We have deleted the Funding Statement from the manuscript.

4. Your ethics statement should only appear in the Methods section of your manuscript. If your ethics statement is written in any section besides the Methods, please move it to the Methods section and delete it from any other section. Please ensure that your ethics statement is included in your manuscript, as the ethics statement entered into the online submission form will not be published alongside your manuscript.

***Response.*** We already had the information from the ethics statement included in the Methods section, so we deleted it from the Statements and Declarations section.

5. We note that Figure 1 in your submission may contain copyrighted images. All PLOS content is published under the Creative Commons Attribution License (CC BY 4.0), which means that the manuscript, images, and Supporting Information files will be freely available online, and any third party is permitted to access, download, copy, distribute, and use these materials in any way, even commercially, with proper attribution. For more information, see our copyright guidelines: http://journals.plos.org/plosone/s/licenses-and-copyright.

We require you to either (1) present written permission from the copyright holder to publish these figures specifically under the CC BY 4.0 license, or (2) remove the figures from your submission:

1. You may seek permission from the original copyright holder of Figure 1 to publish the content specifically under the CC BY 4.0 license.

We recommend that you contact the original copyright holder with the Content Permission Form (http://journals.plos.org/plosone/s/file?id=7c09/content-permission-form.pdf) and the following text:

“I request permission for the open-access journal PLOS ONE to publish XXX under the Creative Commons Attribution License (CCAL) CC BY 4.0 (http://creativecommons.org/licenses/by/4.0/). Please be aware that this license allows unrestricted use and distribution, even commercially, by third parties. Please reply and provide explicit written permission to publish XXX under a CC BY license and complete the attached form.”

Please upload the completed Content Permission Form or other proof of granted permissions as an "Other" file with your submission.

In the figure caption of the copyrighted figure, please include the following text: “Reprinted from [ref] under a CC BY license, with permission from [name of publisher], original copyright [original copyright year].”

***Response.*** We have added three proof of granted permissions to our submission and details about CC license to figure captions.

2. If you are unable to obtain permission from the original copyright holder to publish these figures under the CC BY 4.0 license or if the copyright holder’s requirements are incompatible with the CC BY 4.0 license, please either i) remove the figure or ii) supply a replacement figure that complies with the CC BY 4.0 license. Please check copyright information on all replacement figures and update the figure caption with source information. If applicable, please specify in the figure caption text when a figure is similar but not identical to the original image and is therefore for illustrative purposes only.

6. Please remove your figures from within your manuscript file, leaving only the individual TIFF/EPS image files, uploaded separately. These will be automatically included in the reviewers’ PDF.

***Response.*** We have removed all figures files from the manuscript.

If the reviewer comments include a recommendation to cite specific previously published works, please review and evaluate these publications to determine whether they are relevant and should be cited. There is no requirement to cite these works unless the editor has indicated otherwise.


[Note: HTML markup is below. Please do not edit.]

Reviewers' comments:

5. Review Comments to the Author

Reviewer #1: The authors analyzed the hydrophilic toxins in the skin MeOH extract from the frog Colostethus imbricolus (11 specimens) collected in Colombia. Even though the frogs in this genus were previously reported to have paralytic toxins, they did not detected tetrodotoxin and its analogues using LCMSMS. I think that this is important finding. However, I could not find the detection limit and standard curves of TTX and its analogues for the analytical method they used in this manuscript. These descriptions are needed to be clearly shown.

***Response.*** Unfortunately we did not make a method validation for the analysis of TTX in either of the chromatographic methods presented, as the original aim of the study was to separate and identify the metabolites contained in the skin of *C. imbricolus*. Acknowledging this big limitation of our research we have changed “absence” for “not detected” in lines 448, 452, 487, 496 of the document with track changes. Additionally, in the discussion section we have added this statement in lines 582-586: “Unfortunately, the lack of method validation for TTX analysis, as well as the absence of proper detection and quantification limit estimations for the SB-CN system, makes it difficult to determine whether TTX is truly absent in this species or simply undetectable. However, detecting ions with nominal masses that correspond to TTX-analogues (Table 2) but with fragmentation patterns that do not match previously published data [35–38], supports the idea that *C. imbricolus* lacks these compounds.

Minor points
1. “Graphic abstract: Absence of TTX or any tetrodotoxin analogue”Should be revised to “Graphic abstract: Absence of TTX or any TTX analogues”

***Response.*** We changed the graphical abstract to “No detection of TTX or any TTX analogues”

2. Table 2, why the molecular weights of 254.0000, 272.0000 and others are not the exactly accurate molecular weights of these analogues?

***Response.*** We used the parameters used by Rodríguez et al (2017) in the Table 1 on their supplementary material as a reference to create our chromatographic method. In their study, nominal masses were used because the analyses were performed in MRM mode on a triple quadrupole mass spectrometer. When adapting their method to our Q-TOF mass spectrometer, we also used nominal masses with a ±1 u window instead of tracing accurate precursor ions. We acknowledge this limitation and apologize for it; however, this approach still allowed us to effectively adapt the method to our instrument and monitor the target compounds.

Reviewer #2: The manuscript titled “Lack of Tetrodotoxin analogues and individual metabolic profiling of the cryptic frog Colostethus imbricolus” (PONE-D-25-27319) includes a sophisticated series of analyses that allow the authors to investigate the presence of tetrodotoxin (TTX) and other alkaloid metabolites in a tropical dendrobatid frog species that has been proposed to possess TTX. The authors analyses indicate that TTX is not present in skin sample from the species but that a variety of other alkaloids are present. The authors use an interesting approach to catalog and begin to characterize these other natural products which will allow future investigation of this species as well as the natural products present in the species and genus.
The manuscript is well organized and complete. The experimental design of the project is solid, and I think the approach of using targeted and untargeted LC-MS/MS to investigate TTX and other alkaloids is an elegant solution to a tricky problem. The presence/absence of TTX in the genus Colosthethus is a long-term question that is of interest to ecologists, natural product chemists, and conservation biologists. My sense is that reporting negative results (e.g., the absence of TTX) is important and warrants publication. Because the manuscript also includes a more complete and interesting analysis of the metabolome of the species, I think it warrants publication in PLOS ONE.
The methods and results are clear, and the conclusions and discussion of the results are warranted. My training does not allow me to assess the details of their MS/MS analysis (Table 2) or the data analysis of the metabolomic profiling. However, it appears to me that the authors have provided adequate details for their approach to be replicated and/or judged by someone with more direct experience. Other elements of their analysis seem sound, and I am satisfied that their “null” results and absence of TTX in their samples is accurate and not an experimental artifact.
The discussion and conclusions in the manuscript seem warranted and appropriate for the data set. As with the MS/MS analyses by training does not allow me to assess the more granular level discussion of their data analyses but the broad conclusions and associated with the metabolic analyses appear to be accurate and supported by the data. This approach is a useful tool for building a more complete understanding of the chemical ecology of amphibians and was excited to see it included here.
A minor concern of the paper is the introduction and discussion of the origin of TTX. I am not sure that results presented in the manuscript are relevant to the ultimate origin of TTX as discussed in lines 57 to 62. There is still some disagreement about this. If the authors want to include this discussion, I encourage them to also include recent work from the Yotsu-Yamashita lab that suggests the possibility that TTX-bearing salamanders may synthesize their own TTX and at least acknowledge that for some amphibians the issue is still unresolved.

Overall, the manuscript warrants publication with some minor revisions and clean up. I have identified some specific concerns below:
Specific Concerns:
Lines 58-63. See above, this section may not belong in this paper at all but should include recent paper by Kudo from the Yotsu-Yamashita lab that maps a potential synthesis pathway of TTX from salamander skin intermediates that may not require bacteria.

***Response.*** Thank you so much for this interesting suggestion, it is completely true and we added this fact to the introduction (lines 66-67 of the version with track changes).

Lines 70-73. I understand this sentence (I think), but the structure was confusing and could be clarified.

***Response.*** We edited the text: “Then using behavioral mouse bioassays injecting frog skin extracts, the existence of water-soluble toxins was discovered [16]. It took 15 years using HPLC-FLD analysis to separate and identify tetrodotoxin (TTX), anhydroTTX and 4-epiTTX in C. panamensis [12], the first hydrophilic alkaloids found on the superfamiy Dendrobatoidea.”

Line 79. This substance or these substances not as written.

***Response.*** Text edited to “these substances”.

Lines 91-93. Why are the authors only talking about 11-oxoTTX here? There are other analogs (e.g., 6-epiTTX) that also have terrestrial/marine distribution biases. Why discuss one but not the other?

***Response.*** We were more focused on the chromatographic method for that sentence, but we added the major distribution of 6-epi TTX in terrestrial animals too.

Materials and Methods
I thought this section was well written and complete. However, one small request would be to include the molar concentration of their TTX standard in addition to the PPM concentration. It makes it easier for non-chemists to get a sense of detection limits with these approaches.

***Response.*** We have added the equivalency of 10 ppm to ~31 uM

Another question that may represent ignorance on my part is the use of targeted and untargeted with reference to MS/MS analysis. As somebody slightly outside the field it took me a bit to realize what the terms were referencing (difference is MS/MS mode) it might help a broader audience to understand the results and design of the project to clarify why the two different approaches in the introduction.

***Response.*** In lines 283-286 we have added a short definition of each approach. The differences between these approaches are the columns, the chromatographic conditions and the MS/MS settings.

Discussion
Lines 416-419. This sentence was confusing to me “in spite” of what? I was not sure what the first part of sentence was.

***Response.*** We slightly change the writing to show that it is unexpected that the best chromatographic conditions were obtained in the reversed system for an apparently highly hydrophilic secretion, soluble in highly hydrophilic solvent.

One thing that seemed missing from the discussion was explicit confirmation that none of the TTX analogs, potential precursors, and/or TTX metabolites (e.g., anhyrdro-TTX) are present in the metabolomes. Kudo et al. “Structures of N-Hydroxy-Type Tetrodotoxin Analogues and Bicyclic Guanidinium Compounds Found in Toxic Newts” document a range of compounds using MS/MS detection. Are these compounds included on the comparative database or annotations? Can the author’s results be used to confirm that other TTX related compounds are not present in their samples? This may not be possible, but it would be of interest if it could be confirmed.

***Response.*** The first sentence of the discussion mentioned that “employing both normal-phase and reversed-phase chromatography, revealed that neither TTX or TXX-analogues were detected in this species (Fig 2 and S1)”. However, we noticed that Table 2 needed more details about the metabolites traced in the targeted analysis, because we did not trace all TTX analogs, potential precursors, and/or TTX metabolites reported in the literature, just some of them, so que add this information to Table 2. Adding this important information we noticed that even saxitoxin (STX) was traced, so we also added this information, and stated in the results section that neither of the metabolites traced in the targeted analysis was detected in our model species.

Thank you for providing this interesting perspective about the guanidinium compounds. Usually the annotation of the compounds needs to be done using a specific criteria, so we selected only those molecular formulas detected simultaneously on both approaches (targeted and untargeted). We did not trace all the known TTX analogues in the targeted analysis, only those contained in Table 2 following a previous study (Rodríguez et al., 2017), STX and the most abundant ion detected in our own samples. It is possible to trace specific compounds in both approaches by searching their molecular formulas, but it was not the aim of our study. However, following your recommendation we do traced the molecular formulas of bicyclic guanidinium compounds Tgr-238 and Tgr-240 from Kudo et al. (2020) and cyclic guanidine compounds Cep-210, Cep-212, Cep-242, Cep-240, Cpy-240 from Kudo et al. (2016) and did not find them on our untargeted analyses (S3 Table and S4 Table). We acknowledge how sparse is the information about metabolites in amphibians and how desperately we need an updated database to trace hydrophilic (not only lipophilic) compounds in our samples or future samples. However, we decided to not include further details about the absence of molecular ions corresponding to the molecular ions of these guanidinium compounds because there are hundreds of hydrophilic metabolites that have not been compared with our samples, including chiriquitoxin, TTX-8-O-hemisuccinate and 4-S-Cys TTX that are currently missing in our database.

6. PLOS authors have the option to publish the peer review history of their article ([what does this mean?](https://journals.plos.org/plosone/s/editorial-and-peer-review-process#loc-peer-review-history)). If published, this will include your full peer review and any attached files.

If you choose “no”, your identity will remain anonymous but your review may still be made public.

**Do you want your identity to be public for this peer review?** For information about this choice, including consent withdrawal, please see our [Privacy Policy](https://www.plos.org/privacy-policy).

Reviewer #1: No

Reviewer #2: No
