# Supplementary material for: Lack of tetrodotoxin analogues and individual metabolomic profiling of the cryptic frog Colostethus imbricolus
Source: PLoS One. 2026 Apr 16;21(4):e0325877. doi: 10.1371/journal.pone.0325877 (PMC13086437; doi:10.1371/journal.pone.0325877)
Supplement: Attachment — Submitted filename: Response reviewers- PLOSONE-Colostethus2.docx [file pone.0325877.s010.docx]

**Review Comments to the Author**

Please use the space provided to explain your answers to the questions above. You may also include additional comments for the author, including concerns about dual publication, research ethics, or publication ethics. (Please upload your review as an attachment if it exceeds 20,000 characters)

Reviewer #2: (No Response)

Reviewer #3: This study investigates tetrodotoxin and other alkaloids in order to identify the paralysis-producing substances present in Colostethus imbricolus. Although the toxin itself was not identified, the detection of TTX and amphibian alkaloids, as well as the chemical profiling of Colostethus imbricolus and the metabolomic comparison between males and females, provide valuable information. However, the following concerns need to be addressed.

First, the statement that “TTX was not detected” simply means that its concentration may have been below the detection limit. The detection limit of TTX under the analytical conditions used in this study must be clearly stated. In addition, it is necessary to spike the frog extract with TTX and confirm that it can be detected without ion suppression. This is especially important because the SB-CN column is being applied for the first time in this analysis.

***Response.*** Unfortunately we did not make a method validation for the analysis of TTX in either of the chromatographic methods presented, as the original aim of the study was to separate and identify the metabolites contained in the skin of C. imbricolus. Acknowledging this big limitation of our research we intentionally used the term “not detected”. Additionally, in the discussion section we openly acknowledged this important limitation of our study stating: “Unfortunately, the lack of method validation for TTX analysis, as well as the absence of proper detection and quantification limit estimations for the SB-CN system, makes it difficult to determine whether TTX is truly absent in this species or simply undetectable. However, detecting ions with nominal masses that correspond to TTX-analogues (Table 2) but with fragmentation patterns that do not match previously published data [35–38], supports the idea that C. imbricolus lacks these compounds.

Abstract: “A notable additional outcome of this study is the first successful separation of TTX on an SB-CN column using a normal-phase gradient, enabling a novel method for TTX-targeted separation.”

To claim novelty and usefulness of the SC-CN column for TTX-targeted analysis, validation of the method—such as detection limits, quantification capability, and reproducibility—is essential. Any statements regarding the novelty or utility of the analytical conditions should be made with caution unless supported by proper validation data.

***Response.*** We really appreciate that you have noticed that this statement is not fully supported for the lack of method validation. We have edited the text to “A notable additional outcome of this study is the first successful separation of TTX on an SB-CN column using a normal-phase gradient, suggesting a potential useful approach for TTX-targeted separation.”

Table 1: The functional groups (R1–R4) should be clearly defined.

***Response.*** We are very sorry for this mistake that was originated from a previous version of the manuscript. We have edited the figure to show exclusively the structure of TTX without R1-R4 functional groups.

Table 2: 1-Hydroxy-5,11-dideoxyTTX was structurally revised in a 2020 Journal of Natural Products paper on TTX in newts, and the previously reported structure is no longer considered valid. The corrected compound name should be used.

***Response.*** We really appreciate that you have noticed that the name was revised. We have edited the name in Table 2 to “1-hydroxy-8-epi-5,11-dideoxyTTX”. We also noticed a typo in the structure 11-norTTX-6(R)-ol and we have corrected this in the same table.
